# Brazilian Insect Survey: A Platform for Pest Management

**DOI:** 10.1007/s13744-026-01426-2

**Published:** 2026-09-23

**Authors:** Telmo De Cesaro Júnior, Bárbara Stella Wehrmann, Alexandre Tagliari Lazzaretti, Roberto Wiest, Jorge Luis Boeira Bavaresco, Brenda Slongo Taca, Nicolas Welfer Kirinus, Crislaine Sartori Suzana-Milan, Jayme Garcia Arnal Barbedo, Douglas Lau, Rafael Rieder

**Affiliations:** 1 Federal Institute of Education, Science and Technology Sul-rio-grandense (IFSul), Passo Fundo, RS Brazil; 2 University of Passo Fundo (UPF), Passo Fundo, RS Brazil; 3 Brazilian Agricultural Research Corporation (Embrapa Florestas), Colombo, PR Brazil; 4 Brazilian Agricultural Research Corporation (Embrapa Agricultura Digital), Campinas, SP Brazil

**Keywords:** Integrated pest management, Computer vision, Entomological monitoring, Precision agriculture, Agricultural decision support systems

## Abstract

Modern agriculture faces significant challenges in integrated pest management, where collecting, connecting, and processing monitoring data in real time are essential. This study presents the Brazilian Insect Survey (BIS), a web-based platform designed to centralize and streamline phytosanitary data management, enabling the integration of computer vision, field experimentation, and population modeling within a unified digital ecosystem. The platform is organized into functional modules that support experimental data management (TrapSystem and AgroExperiment), automated insect detection and counting from digital images (InsectCV and AphidCV), and simulation of aphid population dynamics (ABISM). These components operate in synergy, supported by distributed processing infrastructure, to ensure scalable data handling and efficient analytical workflows. Multi-year field applications demonstrate that integrating field data acquisition and automated image analysis with mechanistic population modeling enables timely, model-driven interventions that reduce aphid infestation levels and protect crop yield potential under variable environmental conditions, providing empirical evidence for queryPlease check if the captured keywords are correct.the effectiveness of combining AI-based insect detection with population dynamics modeling in operational integrated pest management. The case studies presented here demonstrate that the BIS platform successfully integrates computer vision, field experimentation, and population modeling within a modular digital ecosystem, highlighting its potential to enhance decision-making and advance data-driven integrated pest management.

**Supplementary Information:**

The online version contains supplementary material available at https://doi.org/10.1007/s13744-026-01426-2.

## Introduction

Modern agriculture faces increasing challenges in crop protection, as agricultural pests represent a major threat to food security and global food production (Yonbawi et al. 2023; Hechen et al. 2024). In this context, Integrated Pest Management (IPM) stands out as a fundamental strategy that promotes decision-making based on systematic monitoring of insect populations and the integration of multiple control methods. This approach seeks to balance economic costs, environmental impacts, and social implications of pest control interventions while sustaining crop yields (Angon et al. 2023; Ciampi et al. 2023; Zhou et al. 2024).

Within this framework, computational tools that support pest and disease surveillance have become increasingly important. These systems have been associated with improved productivity and reduced operational costs in agricultural management (Anastasiou et al. 2025). By enabling the digitalization and streamlining of data collection, storage, and analysis compared to traditional manual methods, such tools provide more reliable and timely insights for decision-making within IPM programs (Ciampi et al. 2023; Rustia et al. 2023; Manoj et al. 2024).

To accelerate the flow of information between field observations and decision-making processes, several digital platforms for pest and disease management have been developed (Zhang and Long 2025). Illustrating this trend, Forresi et al. (2024) proposed a web-based platform that integrates real-time trap data and environmental variables into interactive dashboards, providing farmers and technicians with tactical and strategic support for pest management. In Brazil, examples include the *Consírcio Antiferrugem*,^1^ which monitors the dispersal of Asian soybean rust nationwide, and *Monitora Oeste*,^2^ which tracks diseases affecting soybean and cotton crops in Bahia. Internationally, the Rothamsted Insect Survey^3^ provides long-term monitoring of insect populations across the United Kingdom. More recently, large-scale public initiatives, such as Portugal’s One Health Vector Network (OHVeNet),^4^ have emerged to implement nationwide smart trap networks powered by computer vision and artificial intelligence (AI) for real-time pest and vector surveillance.

Despite these advances, data acquisition in agriculture, computer vision analysis, and population modeling are often implemented as separate tools with limited interoperability. This fragmentation typically requires manual data transfer between systems, slowing data processing and delaying decision-making in pest management programs (Raj and Prahadeeswaran 2025). Integrating these components into unified digital platforms, therefore, remains an important challenge in the development of next-generation decision support systems for agriculture.

The dynamics of the grass-aphid-BYDV-natural enemy complex in winter small grains present substantial tracking challenges, requiring a comprehensive view of how abiotic factors affect biotic elements and their interactions (Lau et al. 2021). Inspired by initiatives such as the Rothamsted Insect Survey, the Brazilian Insect Survey (BIS) platform was conceived as an integrated solution for tracking insect pests in winter cereal crops.^5^

To address this, a series of independent computational modules, such as TrapSystem, ABISM, AphidCV, and InsectCV, were initially developed to support data acquisition, population assessment, and predictive modeling (Lau et al. 2017, 2022). The platform was subsequently expanded into an integrated portal to unify these diverse tools into a single modular digital ecosystem, streamlining decision-making and advancing data-driven IPM. Its development involved a collaborative effort among Embrapa^6^ Wheat, the Federal Institute Sul-rio-grandense (IFSul), and the University of Passo Fundo (UPF), with computational infrastructure support provided by Embrapa Digital Agriculture. BIS seeks to overcome the fragmentation commonly observed among data acquisition, automated analysis, and population modeling systems by providing a unified environment for data management and decision support (Dawson et al. 2024).

The main contribution of BIS is the integration of data acquisition, automated insect analysis, and population modeling into a single digital ecosystem, reducing information silos and facilitating the transition from raw monitoring data to actionable information. By supporting the entire workflow within a unified environment, the platform contributes to more efficient pest surveillance and decision-making processes in agricultural systems (Borrero and Mariscal 2022).

Unlike most platforms that focus primarily on data collection and visualization, BIS combines entomological monitoring, automated insect analysis, and predictive population modeling within an integrated framework. By linking surveillance, automated analytics, and forecasting capabilities, the platform supports the generation of actionable knowledge from field observations and enables data-driven approaches for pest management and epidemic preparedness (Soubeyrand et al. 2024; Helps et al. 2024).

In this context, this paper presents the BIS platform as an integrated digital infrastructure for digital IPM. The “Materials and Methods” section presents the system architecture and the methodological procedures adopted for system design and evaluation. The “Results” section describes the platform components and their integration, as well as the main functionalities supporting insect monitoring and IPM applications. The “Discussion” section discusses system architecture, AI performance, field validation outcomes, operational implications, and system limitations. Finally, the “Conclusions” section concludes the paper and outlines future research directions.

## Materials and Methods

This section presents the computational architecture of the BIS platform and the methodological procedures adopted for its validation and performance evaluation.

### Platform Architecture and Infrastructure

The BIS platform was designed as an integrated digital ecosystem for phytosanitary monitoring, data management, and computational analysis of insect populations (Rosa Ámd et al. 2024). To ensure scalability, high availability, and efficient processing of heterogeneous datasets, the platform adopts a microservices-based architecture deployed on a distributed computational infrastructure maintained by Embrapa Digital Agriculture.

The infrastructure is organized into specialized virtual servers, each responsible for a specific set of services. This separation isolates computationally demanding tasks from user-facing operations, preventing resource contention and improving overall system responsiveness. The infrastructure comprises:

- a Data Server, responsible for managing the central database and storing large-scale image repositories generated by insect traps;
- a Web Server, which functions as the API gateway and integration layer between the platform interfaces and analytical services;
- an Experimental Management Server, dedicated to processing phenotypic and field experiment data;
- a Probabilistic Modeling Server, responsible for executing simulation and forecasting algorithms, including ABISM module; and
- a GPU Cluster, which provides the computational resources required for deep learning inference performed by the InsectCV and AphidCV modules.

The software architecture follows a service-oriented design in which independent modules communicate through REST-based application programming interfaces (APIs). This approach facilitates system maintenance, enables the independent evolution of individual services, and supports the integration of new analytical modules without affecting the platform’s core functionalities. The use of standardized communication interfaces also promotes interoperability among monitoring, analytical, and modeling services, enabling information generated at different stages of the workflow to be exchanged automatically across the platform.

The user interface was developed using React and React Bootstrap, providing responsive access through desktop and mobile devices commonly employed in laboratory and field activities. Back-end services were implemented using Node.js and TypeScript, enabling structured communication among microservices and supporting the management of biological, geographic, and experimental datasets.

Different storage strategies were adopted according to data characteristics. Structured information, including user records, biological observations, monitoring metadata, and experimental parameters, is maintained in a PostgreSQL relational database. Large unstructured datasets, particularly high-resolution trap images, are stored in a distributed file repository integrated with the platform infrastructure. This separation improves storage efficiency and facilitates the management of large image collections generated during monitoring activities. A centralized data management strategy was adopted to ensure consistency across integrated services, allowing information generated by different platform components to be stored, retrieved, and shared through a unified information layer.

To ensure secure access to platform resources, BIS implements a centralized authentication mechanism based on JSON Web Tokens (JWT). This approach provides controlled access to platform services while supporting secure communication among integrated components and user-facing applications.

BIS was organized as a set of interconnected services, each responsible for specific tasks in pest monitoring and management. By exposing standardized REST endpoints and maintaining clear separation among services, the platform can be deployed in other institutional environments and extended through the incorporation of additional analytical and decision-support tools. This architecture enables the seamless exchange of information among monitoring, analysis, and simulation services, supporting an integrated workflow for pest surveillance and management.

### Automated Image Analysis Evaluation

The automated detection capabilities of the InsectCV module (De Cesaro et al. 2022) were evaluated using image datasets obtained from yellow sticky traps (YSTs) and Moericke traps (pan traps). Within the BIS platform, InsectCV acts as the primary source of image-derived monitoring data, generating standardized records for subsequent integration into the analytical components of the system.

For YST monitoring, image acquisition was performed using a dedicated imaging system designed to standardize capture conditions, including illumination and focal distance. The evaluation dataset for YSTs focused on aphid detection and comprised 314 images containing 276 annotated aphid instances.

For Moericke-trap samples, specimens collected in the field underwent laboratory preparation and were digitized using an automated workflow adapted for samples preserved in aqueous media (De Cesaro et al. 2022). This dataset comprised 1030 images containing both aphids (pests) and parasitoids (natural enemies).

Model performance was evaluated using standard object-detection metrics, including Precision, Recall, and mean Average Precision (mAP). Performance was reported at an Intersection over Union (IoU) threshold of 0.50 (mAP50) and averaged across IoU thresholds from 0.50 to 0.95 (mAP50-95). Complementary analyses included F1-score optimization across confidence thresholds and confusion matrix evaluation to assess class-specific detection rates.

To assess operational applicability and long-term consistency, automated counts generated by the system were compared against manual counts from a 7-year historical monitoring dataset obtained from Moericke traps using the coefficient of determination ($R^2$). Finally, operational efficiency was assessed by measuring the complete processing time per image, including image transfer, inference, and result generation.

### Field Evaluation and Simulation-Driven Management

The field trials were conducted during the 2021, 2022, 2023, and 2024 winter crop seasons at the experimental field of the University of Passo Fundo (UPF), Rio Grande do Sul, Brazil ($28^\circ 13'31^{''}S, \ 52^\circ 23'20^{''}W$). The experiment followed a randomized complete block design with four replicates. Plots measured 18 $m^{2}$ (3.6m x 5 m).

For the purposes of this study, three distinct insecticide management strategies were analyzed:

1. **Untreated Control (SI)**: No chemical intervention was performed, serving as a baseline for natural aphid population growth and peak infestation;
2. **Total Treatment (TT)**: A calendar-based strategy consisting of systemic seed treatment followed by weekly foliar insecticide applications (thiamethoxam + lambda-cyhalothrin) throughout the entire vegetative and reproductive cycles to maintain near-zero infestation levels;
3. **ABISM Treatment**: Decision-making was driven by the Agent-Based Insect Simulation Model (ABISM). Applications were triggered by model-generated alerts that utilized daily thermal sums and environmental data to predict when the population would reach the critical action level.

Field monitoring was performed weekly from plant emergence (July) until physiological maturity (October). In each plot, 18 plants were randomly selected for direct visual inspection to determine the number of aphids and the percentage of infested plants. During the vegetative stages (emergence to booting), the entire plant was inspected; from the heading stage onward, sampling focused specifically on the spikes.

The adopted economic action level (NA) followed the technical recommendations for winter cereals in Brazil. From emergence to booting, the threshold was set at 10% of plants infested with aphids. For the first application of the season, a more conservative threshold of 5% was occasionally used to prevent early-season viral transmission. From heading to the milky grain stage, the threshold shifted to a mean density of 10 aphids per spike/plant. After harvesting in November, grain yield (kg/ha) was determined, measured, and corrected to 13% moisture to evaluate the efficacy of each strategy under varying annual pest pressures.

## Results

This section presents the main modules and workflow of the BIS platform, followed by examples of its practical applicability. A biological case study is also provided to illustrate the use of the system in entomological monitoring and decision-support activities.

### BIS Components and Their Roles in Entomological Monitoring and Decision Support

**Fig. 1:**
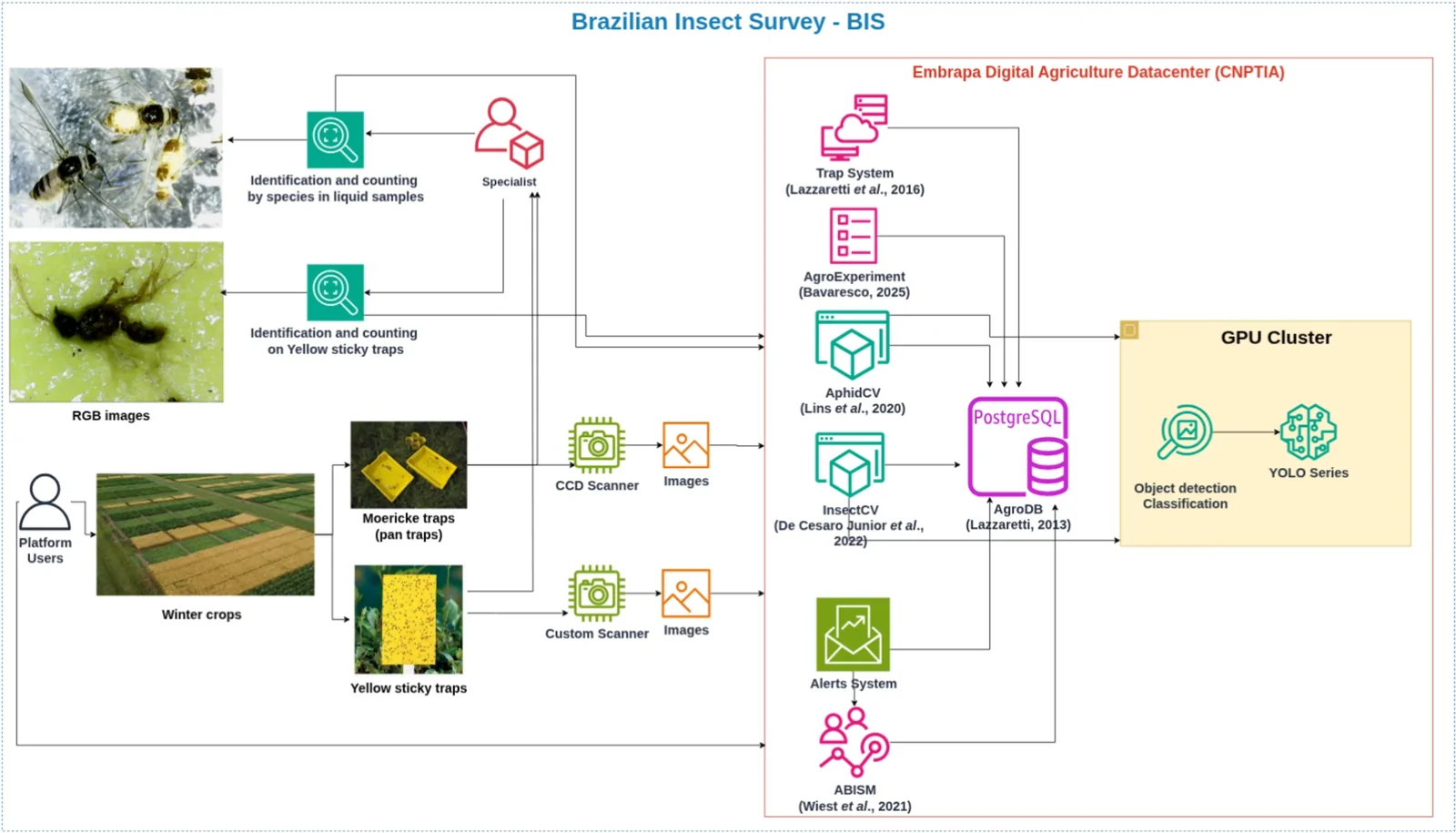
Overview of the BIS ecosystem, illustrating the interactions among modules and the flow of information throughout the platform

The BIS platform integrates complementary tools that collectively support the transformation of entomological observations into information for pest monitoring and decision support. The workflow encompasses field data acquisition, automated image-based analysis, population modeling, and early-warning communication, thereby connecting biological observations with analytical and management activities within IPM programs. An overview of the platform and the relationships among its main components is presented in Fig. 1.

**Fig. 2:**
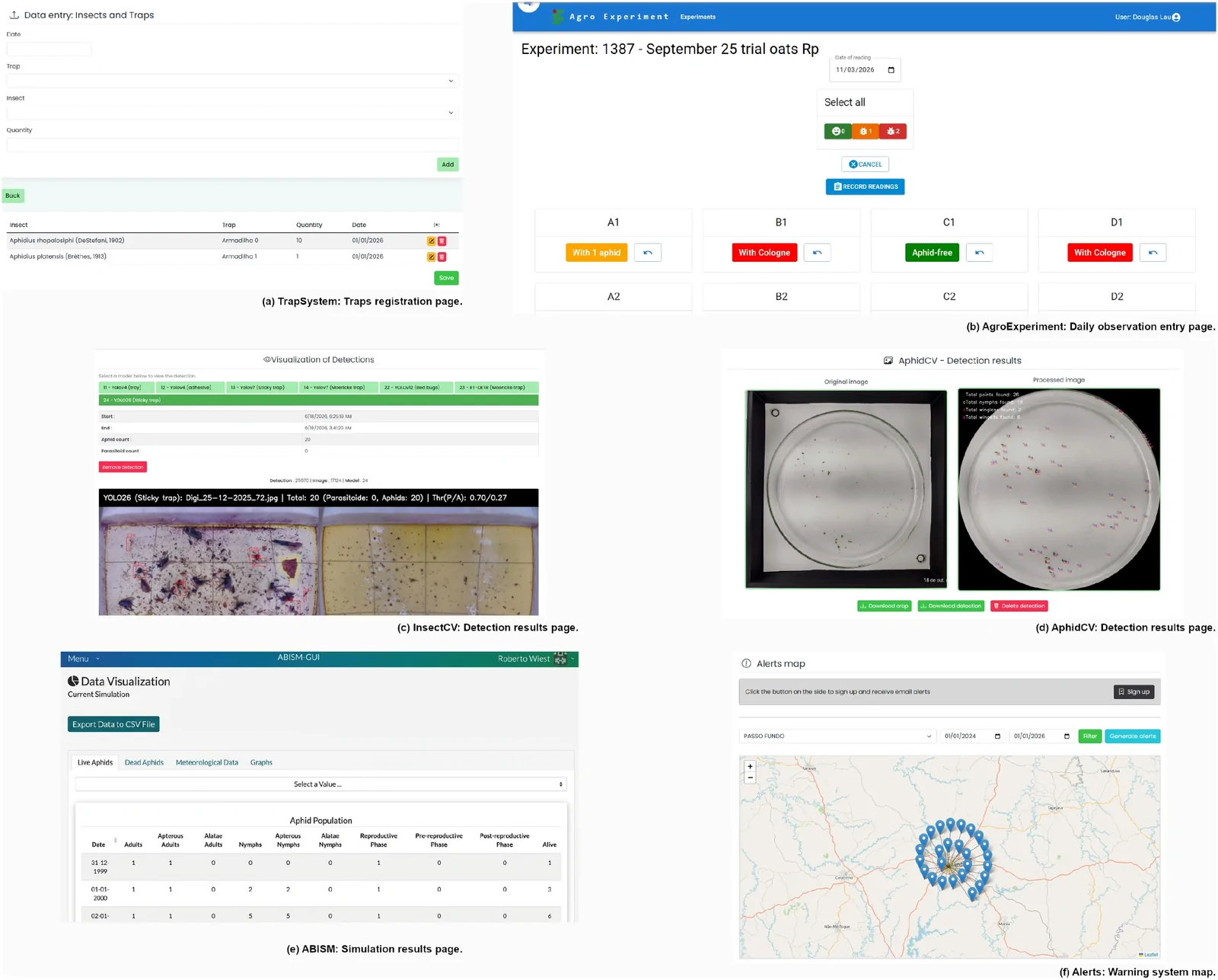
Main analytical and monitoring functions of the BIS platform. From left to right and top to bottom: TrapSystem (monitoring data acquisition), AgroExperiment (experimental observations), InsectCV (automated insect detection), AphidCV (aphid characterization and morphometric analysis), ABISM (population dynamics simulation), and Alerts (information dissemination and decision support)

The main analytical and monitoring functions available within the BIS ecosystem are illustrated in Fig. 2.

#### Data Acquisition and Management

TrapSystem (Lazzaretti et al. 2016b) organizes information obtained from insect-trap networks, including monitoring stations, traps, field surveys, and insect observations. By associating these records with their spatial and temporal context, the module supports the consolidation of monitoring data generated across different institutions and projects. This provides a structured basis for long-term pest surveillance, population analyses, and epidemiological studies (Lau et al. 2021; dos Santos et al. 2022; Engel et al. 2022a, b; Lampert et al. 2023; Lau et al. 2023; Palma et al. 2023; Engel et al. 2025; Palma et al. 2025).

AgroExperiment (Bavaresco and Velasco 2022) complements trap-based monitoring by supporting the structured recording of biological and agronomic observations collected from experimental plots and other experimental settings. By replacing dispersed records with standardized observations, the module improves the consistency and traceability of experimental information collected over time. These observations can be subsequently associated with biological and environmental variables relevant to the analysis of pest population dynamics and predictive modeling.

Together, TrapSystem and AgroExperiment connect routine pest surveillance with controlled biological and agronomic observations, providing complementary sources of information for the BIS workflow. Their integration enables monitoring records and experimental evidence to be jointly used in retrospective analyses, population modeling, and IPM decision support.

#### Automated Image-Based Analysis

Automated image-based analysis addresses one of the main operational limitations of entomological monitoring: the time and labor required for insect identification and counting. While InsectCV is designed for the automated detection and quantification of winged aphids and parasitoids in images from sticky and Moericke traps, AphidCV provides a more granular analysis of pre-identified aphid species. For each species under analysis, the corresponding species-specific model enables the counting of nymphs, apterous adults, and alate adults, as well as the extraction of detailed morphometric data.

InsectCV (De Cesaro et al. 2022; Domênico et al. 2021; Da Silva et al. 2024) processes images obtained from Moericke (pan) traps and yellow sticky traps (YSTs) to automatically detect and quantify insect populations using deep learning techniques. The module supports the processing of large image collections and generates standardized image-derived observations that can be incorporated into the broader BIS monitoring workflow. Additional details on the computational workflow connecting BIS, InsectCV, and the GPU infrastructure are provided in Supplementary Fig. S1. By reducing the dependence on manual counting, InsectCV increases the scalability of insect surveillance and provides quantitative information that can subsequently support population analyses, pest monitoring, and IPM decision-making.

AphidCV (Lins et al. 2020; Rodriguez and Rieder 2020; Kirinus et al. 2023a; Taca et al. 2025b) extends the image-analysis capabilities of BIS by characterizing aphid specimens according to their developmental stage and morphological traits. The module uses species-specific models selected according to the species represented in the analyzed sample, enabling the automated extraction of biological information from laboratory images. Similar to InsectCV, AphidCV is integrated into the BIS image-analysis workflow, allowing the resulting information to be incorporated into the platform’s broader monitoring and analytical processes.

#### Predictive Modeling

The information generated through monitoring and image-based analyses can be further used for predictive population modeling through the Agent-Based Insect Simulation Model (ABISM) (Wiest et al. 2021). By integrating biological observations, monitoring records, and environmental information, ABISM simulates insect population dynamics under different environmental and management conditions.

ABISM enables researchers to explore hypothetical scenarios and evaluate the potential impacts of environmental and management factors on pest population development. By combining observational and simulated information within an integrated workflow, BIS extends the role of digital monitoring systems beyond data storage and visualization, supporting analytical and forecasting activities relevant to IPM programs.

#### Information Dissemination and Alerts

The predictive outputs generated by ABISM can be translated into actionable information through the Alerts module. When predicted pest abundance exceeds a predefined economic action threshold, such as the 10% infestation threshold used for aphids in wheat, the system generates alerts and risk information to support timely management decisions. This connection between population forecasting and management communication is essential for translating monitoring and modeling outputs into operational IPM actions.

#### Integrated Workflow

The main contribution of BIS is the integration of complementary sources of biological information into a continuous workflow for monitoring, analysis, prediction, and decision support. Field and experimental observations collected through TrapSystem and AgroExperiment can be complemented with automated insect detection and characterization provided by InsectCV and AphidCV. These data can subsequently support population simulations through ABISM, whose outputs can be translated into early-warning information through the Alerts module. AgroDB provides the shared data environment required to connect these information streams and maintain the continuity of information across the workflow (Lazzaretti et al. 2016a).

This integration enables BIS to transform heterogeneous biological observations into standardized information for research, pest surveillance, and IPM decision support, while also providing a framework that can incorporate additional monitoring and analytical approaches as required by different agricultural scenarios. As illustrated in Fig. 1, field observations, experimental data, automated image analyses, simulation models, and early-warning communication services interact to form a unified workflow.

### Performance and Practical Applicability of BIS Modules

The operational validation of the BIS platform is based on an integrated workflow that begins with passive trap deployment in the field and culminates in predictive simulations of population dynamics. To demonstrate the practical applicability of this ecosystem, the results are presented following the flow of information across the platform’s integrated modules, highlighting how data are transformed into analytical and decision-support outputs.

#### Image Acquisition and Primary Detection: InsectCV Module

The InsectCV module was developed to automate insect detection and counting from trap images, reducing the time and effort traditionally associated with manual assessments (De Cesaro et al. 2022). Within the BIS workflow, it generates standardized image-derived records that can be integrated with analytical and predictive components of the platform.

For aphid detection in YST images using the YOLO26L model (Jocher et al. 2026), InsectCV achieved a Precision of 0.795, Recall of 0.762, mAP50 of 0.827, and mAP50-95 of 0.500 for aphid detection using the YOLO26L model (Jocher et al. 2026).

For Moericke-trap images, automated detection using the RT-DETR model (Lv et al. 2023) obtained an overall Precision of 0.942, Recall of 0.788, mAP50 of 0.835, and mAP50-95 of 0.440 across the 1030 evaluated images. Reliable detection performance was observed for both pests and natural enemies: aphids were detected with 92.6% Precision (82% correct detection rate in confusion matrix analysis), while parasitoids achieved 95.8% Precision (86% correct detection rate). The overall F1-score peaked at 0.86 at an optimal confidence threshold of 0.496.

Comparison between automated and manual counts from the 7-year historical Moericke-trap dataset demonstrated strong agreement ($R^2 = 0.898$), confirming the reliability of the system for long-term population surveillance (De Cesaro et al. 2022).

Complete image processing, including image transfer, object detection, and data logging, required approximately 12 s per image. This processing time substantially reduces manual workload and enables large-scale IPM monitoring.

#### Characterization and Morphometry: AphidCV Module

AphidCV processes laboratory images of aphid samples using species-specific models selected according to the species under analysis. The current version supports six economically important species: *Rhopalosiphum padi*, *Schizaphis graminum*, *Metopolophium dirhodum*, *Sitobion avenae*, *Myzus persicae*, and *Brevicoryne brassicae* (Kirinus et al. 2023a, b; Taca et al. 2025b). The module characterizes specimens according to developmental stage and extracts morphometric traits, generating quantitative biological information from image data.

Previous validation studies demonstrated strong agreement between AphidCV and specialist-based assessments for aphid counting, developmental-stage classification, and morphometric estimation (Lins et al. 2020). These findings support the reliability of the module for generating standardized quantitative biological information from images.

Previous evaluations also demonstrated a substantial reduction in processing time compared with conventional manual inspection procedures (Rodriguez and Rieder 2020). By increasing sample-processing capacity while preserving biologically relevant information, AphidCV facilitates the incorporation of detailed aphid data into ecological studies, population analyses, and IPM programs.

#### Experimental Management and System Parameterization: AgroExperiment Module

Biological experiments conducted under field and greenhouse conditions provide essential information for understanding aphid population dynamics and their responses to environmental conditions. AgroExperiment (Bavaresco and Velasco 2022) complements trap-based monitoring by supporting the structured recording of biological and agronomic observations collected from experimental units, including field plots and greenhouse assays.

The practical contribution of AgroExperiment lies in the standardization of longitudinal observations collected during biological and agronomic experiments. The module supports the recording of plant development, aphid colonization, and other experimental observations over repeated assessments, thereby improving the continuity and consistency of datasets used in population studies. Previous applications have also demonstrated the feasibility of recording observations directly during field activities, supporting more continuous data collection throughout the crop cycle (Velasco and Bavaresco 2021).

The biological and environmental observations generated during experiments can subsequently be incorporated into predictive analyses. Variables relevant to population dynamics, including temperature, photoperiod, humidity, soil characteristics, and meteorological conditions, can be associated with experimental observations and used to parameterize or inform ABISM simulations. This connection between experimental observations and predictive modeling strengthens the use of BIS as an integrated framework for linking biological measurements, population analysis, and IPM decision support.

**Fig. 3:**
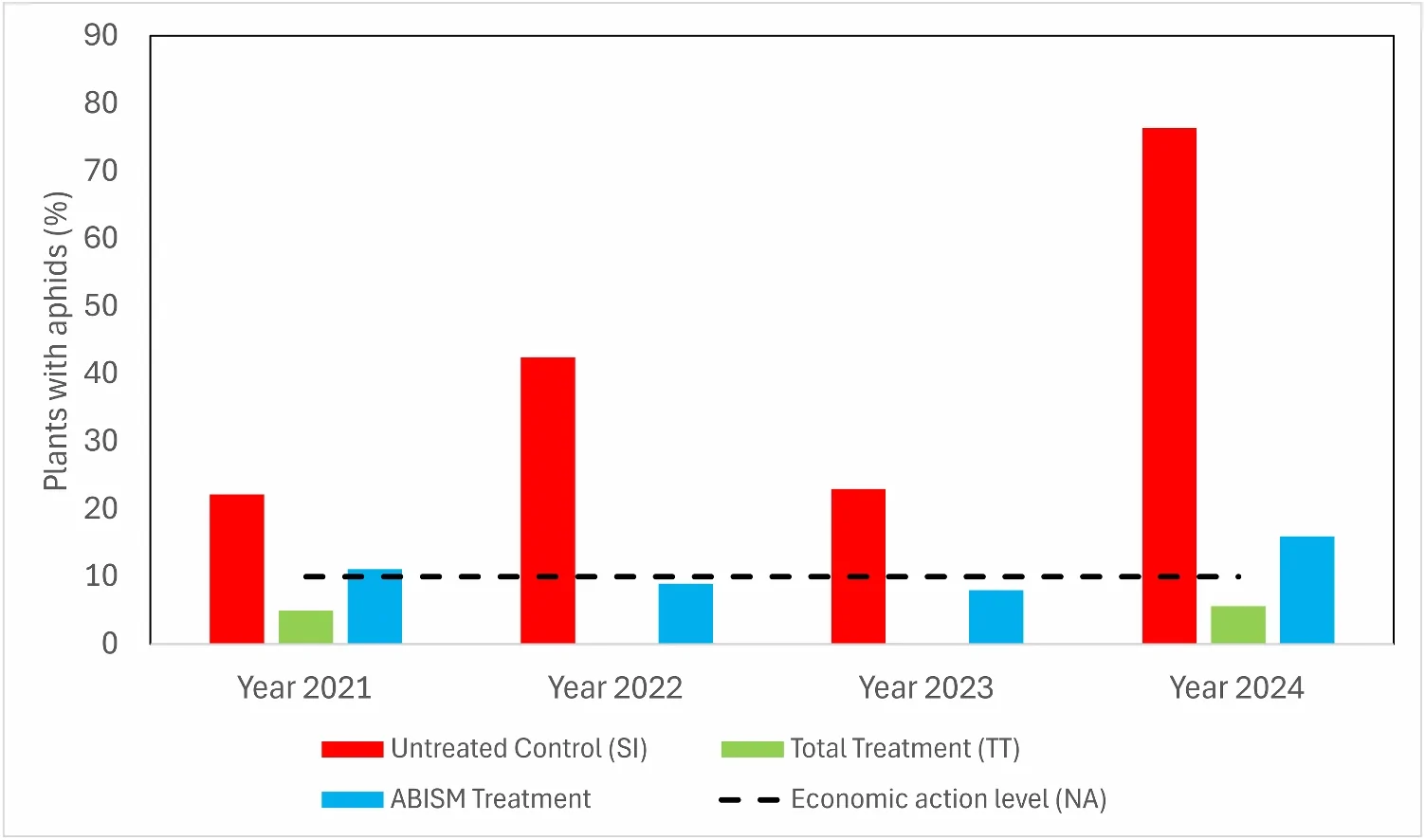
Percentage of plants infested with aphids (Plants with aphids, %) across four consecutive growing seasons (2021–2024) under three management strategies: Untreated Control (SI, red bars), Total Treatment (TT, green bars), and ABISM Treatment (blue bars). The dashed black line indicates the economic action threshold (Economic action level, NA = 10%)

#### Predictive Modeling and Population Dynamics: ABISM Module

ABISM is the predictive modeling component of BIS and extends the platform beyond the collection and analysis of monitoring data by enabling the simulation of insect population dynamics under different environmental and management conditions (Wiest et al. 2021). By combining biological observations, environmental variables, and monitoring records, the model supports the investigation of how insect populations may respond to changing conditions and the evaluation of alternative management scenarios before their implementation in the field (Maurer et al. 2026).

The predictive performance of ABISM has been extensively evaluated using the aphid *R. padi*, one of the main vectors of barley yellow dwarf virus (BYDV) in winter cereals (Wiest et al. 2021). Validation experiments were conducted under constant temperatures, cyclic thermal regimes with amplitudes of up to $10^{\circ }$C, and naturally fluctuating outdoor conditions. Across these scenarios, the model successfully reproduced population dynamics and demographic structure, including the abundance of nymphs, apterous adults, and alate adults.

In a previous validation study conducted under variable outdoor temperature conditions, simulated and observed populations showed strong agreement, with a Root Mean Squared Logarithmic Error (RMSLE) of 0.6 for total population estimates (Wiest et al. 2021). The model also accurately represented the influence of thermal accumulation on aphid development, longevity, and fecundity, supporting its application in studies of population ecology and pest forecasting.

**Fig. 4:**
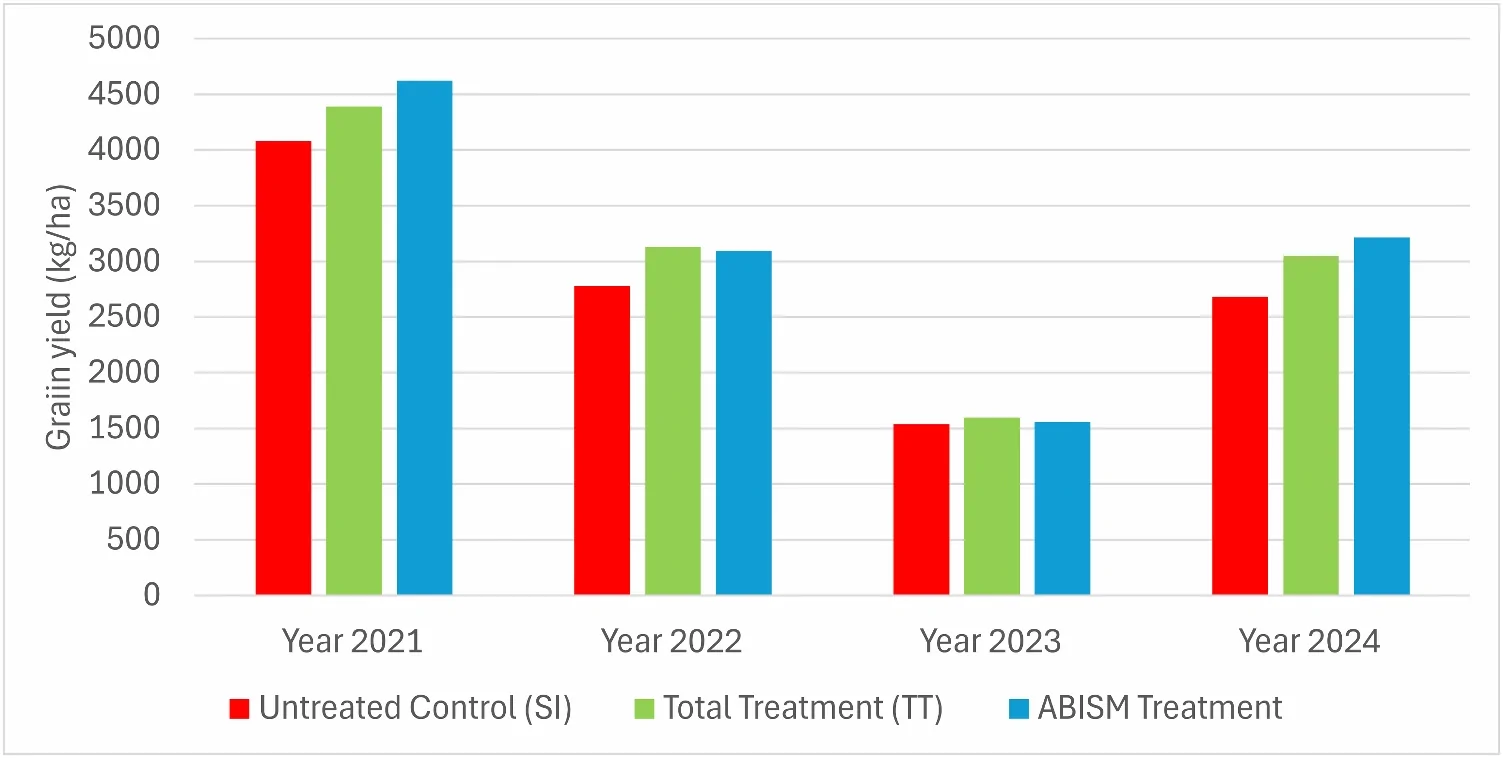
Grain yield ($kg \cdot ha^{-1}$) across four consecutive growing seasons (2021–2024) under three aphid management strategies: Untreated Control (SI, red bars), Total Treatment (TT, green bars), and ABISM Treatment (blue bars)

More recently, ABISM has demonstrated applicability as a predictive tool for the spatiotemporal dynamics of *R. padi* by integrating individual-level biological parameters with environmental thermal regimes. The model effectively captures population progression, supporting the validity of its core simulation logic for field-level warning systems (Maurer et al. 2026). However, its predictive accuracy is influenced by the “host plant effect,” which acts as a primary biological filter for pest proliferation. While the current model calibration closely aligns with infestation patterns in resistant cultivars, it tends to underestimate population growth and spatial colonization in highly susceptible hosts. To address these discrepancies, refinements such as the integration of cultivar-specific resistance classes and the transition to stochastic mortality rules have been proposed. These limitations also highlight the importance of integrating experimentally derived biological information into predictive frameworks. Despite the need for further genotype-specific parameter adjustments, ABISM provides a relevant basis for automated decision support and yield-loss mitigation within IPM systems, as further demonstrated by the field validation presented in the “Validation of Simulation-Driven Management Under Field Conditions” section.

### Validation of Simulation-Driven Management Under Field Conditions

Across the four evaluated crop seasons (2021–2024), the Total Treatment (TT) consistently maintained aphid populations at near-zero levels, with peak infestations never exceeding 5.6% of plants. In contrast, the Untreated Control (SI) exhibited significant annual variation in pest pressure. Peak infestation levels in the SI plots reached 22.2% in 2021, 42.5% in 2022, 23.0% in 2023, and a critical high of 76.4% in 2024 (Fig. 3).

The ABISM-driven management demonstrated proactive control efficacy across all years. In 2021 and 2022, ABISM alerts triggered applications that stabilized peak infestations at 11.1% and 9.0%, respectively. During the high-pressure 2024 season, while the SI treatment peaked at over 76%, the ABISM strategy successfully limited infestation to a mean of 16.0%. In 2023, environmental factors (excessive rainfall) naturally suppressed populations, with ABISM maintaining levels at 8.0% compared to 23.0% in the control.

Grain yield results showed that the impact of management strategies was strongly influenced by annual pest pressure and climatic conditions. From 2021 to 2023, no statistically significant differences (p>0.05) in grain yield were observed among the SI, TT, and ABISM strategies. In 2021, yields were recorded at 4081 kg/ha (SI), 4388 kg/ha (TT), and 4621 kg/ha (ABISM). In 2022, yields were 2781 kg/ha (SI), 3130 kg/ha (TT), and 3095 kg/ha (ABISM). The 2023 season, characterized by El Niño-driven excessive rainfall, resulted in lower and statistically similar yields across all treatments: 1539 kg/ha (SI), 1600 kg/ha (TT), and 1558 kg/ha (ABISM) (Fig. 4).

However, a statistically significant differences was detected in 2024 season was detected in the 2024 season (p<0.05). Under high aphid pressure, the ABISM strategy (3217 kg/ha) and the TT strategy (3048 kg/ha) significantly protected yield potential compared to the SI treatment (2682 kg/ha).

## Discussion

This section examines the architectural and AI performance of the BIS platform, its operational and strategic contributions, the outcomes achieved through field validation, and the main limitations that guide future developments.

### System Architecture and AI Performance

The development of BIS and its constituent modules, including TrapSystem (Lazzaretti et al. 2016b) and InsectCV (De Cesaro et al. 2022), revealed challenges commonly associated with large-scale digital agriculture platforms. As the ecosystem evolved to integrate field monitoring, image analysis, and predictive modeling within a unified environment, maintaining data consistency, interoperability, and efficient management of large image repositories became critical requirements. Similar challenges have been reported in Agriculture 4.0 infrastructures, where the increasing volume and diversity of digital information demand robust mechanisms for data management and scalable storage solutions (Debauche et al. 2022; Macpherson et al. 2022). The adoption of distributed data management strategies enabled BIS to support large-scale image analysis while maintaining the responsiveness required for operational monitoring.

Ensuring secure communication among distributed services represented an additional architectural challenge. Contemporary digital agriculture platforms increasingly rely on cloud-based and service-oriented infrastructures, where interoperability must be balanced against cybersecurity requirements and institutional data governance policies (Hannousse and Yahiouche 2021). Moreover, communication across distributed web services introduces additional security risks and trust dependencies that require careful control of interactions among system components (Chen et al. 2018). Collectively, the architectural refinements implemented in BIS enabled the consolidation of previously fragmented tools into a cohesive digital ecosystem, providing the foundation for advanced analytical tasks such as automated monitoring, temporal trend analysis, ecological forecasting, and decision support, which are increasingly recognized as essential capabilities of next-generation agricultural information systems (Rossi et al. 2023; Tonle et al. 2024).

Beyond the architectural aspects, the effectiveness of BIS ultimately depends on the reliability of its AI-driven monitoring components, since automated insect detection constitutes the primary source of biological information feeding subsequent forecasting and decision-support processes. Comparative experiments were therefore conducted using two state-of-the-art computer vision architectures, YOLO26 and RT-DETR. The adoption of these frameworks is aligned with recent evidence indicating that Deep Learning approaches, particularly models from the YOLO family and Vision Transformer-based architectures, currently represent the state of the art for automated insect recognition and counting in digital images (De Cesaro et al. 2026).

Performance analyses revealed a degree of specialization according to the characteristics of the acquisition process. While YOLO26 achieved the best results for images obtained from YSTs, the RT-DETR architecture demonstrated superior performance when processing more challenging samples acquired from Moericke traps and preserved in liquid media. These findings indicate that the effectiveness of computer vision models in entomological monitoring depends not only on the model architecture itself but also on the visual characteristics of the target dataset (Gao et al. 2024; Hu et al. 2025).

This observation reinforces a growing consensus in the computer vision literature that model performance is strongly influenced by acquisition conditions, image quality, and domain-specific visual characteristics. Consequently, the direct transferability of models across distinct monitoring scenarios remains limited, often requiring dataset-specific adaptation and validation procedures. For agricultural monitoring applications, these results highlight the importance of evaluating AI models under realistic operational conditions rather than relying solely on benchmark datasets (Popescu et al. 2023).

The observed performance suggests that the integration of these models into BIS is sufficiently robust to support large-scale automated monitoring while maintaining the detection reliability required for operational IPM applications. Furthermore, the strong performance achieved for both pest species (aphids) and natural enemies (parasitoids) highlights the potential of the platform to support the automated extraction of biologically meaningful indicators, including measures associated with biological control. By providing reliable and standardized population metrics, the platform optimizes field sampling efficiency and enhances the precision of action thresholds within IPM programs (Marinko et al. 2024; Tonle et al. 2024).

Beyond detection accuracy, the practical value of these models emerges from their integration into a broader decision-support system. Recent studies have emphasized that the successful adoption of AI in agriculture depends not only on predictive performance but also on the ability to transform analytical outputs into actionable information that supports operational management decisions and precision crop protection strategies (Mesías-Ruiz et al. 2023). In this regard, BIS extends the role of computer vision beyond automated insect identification by directly connecting monitoring outputs to forecasting and decision-support components.

More broadly, these results reinforce the importance of integrating modern computer vision techniques into digital agricultural infrastructures. Rather than functioning solely as isolated image-analysis tools, the detection models incorporated into BIS become part of an integrated analytical platform that connects data acquisition, automated processing, information management, and predictive modeling. This integration expands the practical value of AI applications in agriculture, transforming image-derived observations into actionable information that supports monitoring, forecasting, and decision-making within modern IPM programs.

### Strategic Contributions and Operational Implications

The implementation of BIS fills a gap in the digitization of IPM in Brazil by proposing a scalable and flexible modular ecosystem. Unlike initiatives such as *Consórcio Antiferrugem*, which focus on the dissemination of information related to a specific disease, or *Monitora Oeste*, aimed at regional pest monitoring in cotton and soybean production systems, BIS was conceived as a unified computational environment capable of integrating multiple stages of the phytosanitary monitoring process. The platform is conceptually grounded in the Rothamsted Insect Survey, one of the most influential long-term aphid monitoring initiatives worldwide (Crossley et al. 2021), while extending this paradigm through the consolidation of previously independent computational tools into a single ecosystem.

The strategic value of BIS lies in its ability to integrate data collection, image processing, information management, and predictive modeling within a unified digital ecosystem. This architecture reduces data redundancy, standardizes system interfaces, and accelerates the transformation of raw field observations into actionable decision-support information. For example, field records managed through AgroExperiment can be directly incorporated into the ABISM forecasting framework, while population estimates generated through computer vision tools provide complementary information for validating and refining outbreak predictions. Together, these capabilities enable a more comprehensive assessment of crop phytosanitary conditions and strengthen decision-making processes within IPM programs.

From a computational perspective, the architecture based on containerized microservices combined with React’s client-side rendering provides high scalability and a responsive user experience, even when handling large volumes of agronomic data (Taca et al. 2025a). The ability to asynchronously delegate computationally intensive AI tasks to a GPU cluster ensures that the platform remains responsive while supporting large-scale automated analyses, overcoming performance and accessibility limitations commonly associated with monolithic image-processing systems (Li et al. 2024). At the same time, the dependence on a robust institutional datacenter infrastructure introduces challenges related to network management, firewall orchestration, and secure communication among distributed services, which are common concerns in software-defined environments (Kreutz et al. 2014).

Although the current implementation focuses on winter cereals and on the identification of aphids and their natural enemies, for which the deep learning models were trained and validated (De Cesaro et al. 2022), the modular architecture was intentionally designed to facilitate future expansion. New analytical tools, capture methodologies, and target pest groups can be incorporated into the ecosystem without major modifications to the platform core. Nevertheless, extending BIS to additional crops and insect orders will require substantial efforts in data acquisition, expert annotation, and model retraining. These characteristics position BIS not merely as a collection of digital tools, but as an evolving infrastructure capable of adapting to emerging IPM demands and future advances in AI.

### Current State of the BIS Platform and Achieved Milestones

The operational evolution of the BIS platform highlights a successful transition from traditional, labor-intensive pest scouting to automated, simulation-driven precision management. At its core, the platform leverages the ABISM, originally parameterized by Wiest et al. (2021) under constant temperature regimes, to translate individual-level biological parameters into macro-scale field alerts.

The effectiveness of this simulation-driven approach was confirmed through four consecutive years of field validation (2021–2024). By accurately processing daily thermal sums and population parameters, the platform demonstrated sufficient predictive lead time to trigger precise insecticide applications. This optimization successfully suppressed aphid proliferation before economic injury levels were breached, preserving winter cereal yields, preventing losses exceeding 530 kg/ha in high-pressure years such as 2024, and demonstrating the commercial and ecological viability of the ecosystem within modern IPM frameworks.

The successful integration of ABISM into BIS extends the platform beyond conventional retrospective monitoring by enabling users to explore prospective infestation scenarios. Rather than relying exclusively on current insect abundance, researchers and pest management practitioners can anticipate population growth trends, assess the potential influence of environmental conditions, and identify periods of elevated infestation risk. This predictive capability enhances the strategic value of BIS as a decision-support platform, allowing preventive rather than reactive management within IPM programs.

Taken together, the 4-year field evaluations (2021–2024) provide a proof of concept for simulation-driven precision management, demonstrating how the integration of ABISM with the BIS monitoring infrastructure can translate biological observations into timely management actions. By connecting entomological monitoring, automated image analysis, and mechanistic population modeling within a unified ecosystem, BIS extends conventional surveillance beyond retrospective data collection toward a more integrated, data-driven approach to pest management. This integration establishes a foundation for increasingly automated early-warning capabilities, with the potential to improve the timing and precision of insecticide applications while preserving yield potential under variable environmental conditions.

### Weaknesses and Key Areas for System Refinement

Despite these substantial advancements, bridging the gap between controlled laboratory parameters and spatiotemporal field dynamics has revealed critical boundaries. As systematically demonstrated by Maurer et al. (2026), the primary limitation of the current framework lies in its high sensitivity to the “host plant effect.” While the default parameters inherited from Wiest et al. (2021) align closely with the infestation patterns of resistant wheat materials, they significantly underestimate risk, population growth rates, and spatial colonization velocities when deployed over high-performance susceptible hosts.

According to Maurer et al. (2026), this predictive deficit stems from neglecting intraspecific cultivar variations and insect-plant interactions. High-performance susceptible hosts drastically alter the aphid’s life-history traits at the individual level by shortening the pre-reproductive period while substantially increasing total fecundity and extending longevity, thereby accelerating field-wide colonization. Because the original ABISM architecture treats host plants uniformly, it fails to capture how morphological traits (e.g., the presence or absence of trichomes acting as physical barriers) or phloem nutritional quality filter pest proliferation.

To overcome these boundaries and advance the platform’s predictive maturity, future refinements must prioritize three strategic areas:

- **Integration of Cultivar-Specific Resistance Classes**: Transitioning from generalized host profiles to standard, JSON-mapped resistance groups (e.g., susceptible vs. resistant classes) that dynamically adjust development and natality scales within the “Pest” and “Field” modules based on the specific crop genotype sown;
- **Transition to Stochastic Mortality Rules**: Replacing the current threshold-based, deterministic mortality logic, in which all aphid agents die abruptly upon reaching a predefined thermal sum, with dynamic daily survival probabilities that model cumulative risk and natural senescence. This structural fix, as recommended by Maurer et al. (2026), is essential to prevent artificial population crashes and accurately simulate overlapping insect generations on high-performance hosts;
- **Synergistic Host-Vector-Pathogen Modeling**: Expanding the automated decision-support matrix beyond simple aphid population thresholds by systematically integrating the specific interaction between host-vector resistance and host-pathogen tolerance (for example, BYDV classes) to accurately mitigate final grain yield losses under fluctuating field environments.

## Conclusions

This study introduced BIS, an integrated digital platform that unifies entomological monitoring, automated image analysis, and mechanistic population modeling into a continuous data-to-decision pipeline for winter crop IPM. Multi-year field validation demonstrated that coupling real-time data with the ABISM simulation model enables timely, model-driven interventions that consistently reduce aphid infestations and preserve grain yields, even under high pest pressure. Driven by deep learning, the platform’s high-throughput capability successfully bridges the gap between heterogeneous biological data and actionable field operations. Future work will enhance the ecological realism of the ABISM model, extend its framework to additional cropping systems, and fully automate the early-warning decision cycle through real-time predictive alerts.

## Supplementary Information

Below is the link to the electronic supplementary material.

Supplementary file 1 (tex 0 KB)

## Footnotes

1 Available at http://www.consorcioantiferrugem.net.

2 Available at https://mapas.cnpm.embrapa.br/apps/monitora_oeste.

3 Available at https://insectsurvey.com.

4 Available at https://tinyurl.com/Portugal-OHVeNet.

5 Available at https://www.bis.cnptia.embrapa.br.

6 The Brazilian Agricultural Research Corporation.

## Declarations

### Software Availability

Software name: BIS - Brazilian Insect Survey Developers: Embrapa Wheat, IFSul, and UPF Contact address: BR 285, Km 292.7, Passo Fundo, RS, Brazil. CEP 99052-900 Email: telmojunior@ifsul.edu.br Year first available: 2024 Hardware required: Production-grade web server for application hosting and a dedicated NVIDIA GPU cluster for server-side inference Software required: Standard web browser for client access; PostgreSQL database (AgroDB), Docker, and a Node.js runtime environment Programming languages: TypeScript (React, Express), JavaScript, and HTML Availability and access: The platform is publicly available and can be accessed at https://www.bis.cnptia.embrapa.br. Source code: The source code is hosted on GitHub across private repositories. Due to the institutional security and intellectual property policies of the partner institutions, full access is currently restricted to authorized developers. However, access for peer review purposes or scientific collaboration can be granted upon formal request to the corresponding author.
